# Commentary: The entropic brain: a theory of conscious states informed by neuroimaging research with psychedelic drugs

**DOI:** 10.3389/fnhum.2016.00423

**Published:** 2016-08-30

**Authors:** David Papo

**Affiliations:** GISC and Laboratory of Biological Networks, Center for Biomedical Technology, Universidad Politécnica de MadridMadrid, Spain

**Keywords:** metastability, scaling, criticality, entropy, brain activity, temporal complexity

The “entropic brain hypothesis” holds that the quality of conscious states depends on the system's entropy (Carhart-Harris et al., 2014). Brain activity is said to become “more random and so harder to predict in primary states – of which the psychedelic state is an exemplar.” Psychedelic-induced brain activity would be associated with elevated entropy in some of its aspects with respect to normal wakeful consciousness. This would indicate that psychedelic-induced brain activity would exhibit criticality, while normal wakeful consciousness would be subcritical.

But can *entropy* be a unique indicator of the “quality of consciousness?” Are there reasons to believe that psychedelic-induced activity is *not* critical?

## Entropy

Complex systems such as the brain are neither completely regular nor completely random. Entropy a measure of randomness, may then seem ill-qualified, *per se*, to account for brain activity. Indeed to account for the complex blend of regularity and randomness, a wealth of so-called complexity measures have been proposed (Crutchfield, 2012), some specifically for brain activity (Tononi et al., 1994; Tognoli and Kelso, 2014). A single-scale indicator of randomness to describe the consciousness spectrum simply boils down to a complexity measure depurated of its integration or disequilibrium term.

Efforts to characterize brain states, and consciousness in particular, in terms of entropy of the associated dynamics have a 20-year long history. For instance, a significant reduction in EEG entropy (Pezard et al., 1996), and co-variation between entropy and mood improvement were found in depressive patients (Thomasson et al., 2000). Furthermore, entropy monitoring is now a standard method to evaluate the depth of anesthesia (Bein, 2006; Jordan et al., 2008; Olofsen et al., 2008).

## Metastable states and metastability

A system is metastable when it spends an extended time in a configuration other than the system's least energy state (Kitzbichler et al., 2009; Allegrini et al., 2010; Tognoli and Kelso, 2014). Activity shows a *dwell* and *escape* dynamics: *Within* a metastable state, physical quantities, e.g., energy, fluctuate reversibly around a constant value, while the escape part of the dynamics is associated with shifts from one metastable state to another. Metastability is characterized in terms of dynamical variables, e.g., avalanche size and duration, or inter-burst intervals.

Carhart-Harris and colleagues define metastability as a “measure of the variance in the network's intrinsic synchrony” (p. 10). However such a measure quantifies (the number of) *metastable states* (Haldeman and Beggs, 2005; Shanahan, 2010), rather than *metastability*, so that entropy, metastability, and variance turn out to provide essentially the same information. Carhart-Harris' entropy is a *configurational entropy*, which essentially counts the metastable states of the energy landscape. A genuine configurational entropy establishes a connection between the topography of the energy landscape and the system's dynamics. However, the coarse-graining level must be drastic for one to claim that a few hundred time-points (Carhart-Harris et al., 2012) sufficiently explore the brain's phase space, (how the number of metastable states scales with coarse-graining level is also unknown). Thus, the associated entropy cannot be considered a genuine configurational entropy.

## Psychedelic state and criticality

Criticality of the psychedelic state is inferred through the following reasoning: Criticality maximizes the amount of metastable states (Haldeman and Beggs, 2005), and therefore entropy; psychedelic states are associated with more metastable states than normal wakefulness; therefore, the corresponding state is critical, while normal wakefulness is subcritical (Priesemann et al., 2014).

Even from a complexity view-point, some value of the configuration entropy may well correspond to brain activity patterns for which the system is critical. However, there are reasons to doubt that psychedelic-associated activity is critical. (1) Criticality implies (a set of) scaling variables, e.g., avalanche size and duration. Nothing guarantees that the dynamics producing these metastable states in Carhart-Harris et al. (2014) would also generate a dynamics showing scaling as in Haldeman and Beggs (2005). Furthermore, the few hundred timepoints in Carhart-Harris' data (Carhart-Harris et al., 2012) are insufficient to show temporal scaling behavior. (2) While Carhart-Harris' data show that the psychedelic state is associated with more variance than normal wakefulness, other states could in principle be associated with yet more variance than the one associated with the psychedelic state. Furthermore, increasing variance from a subcritical state doesn't guarantee that the system will become critical or that it will have a better power-law fit. (3) Recent evidence shows that, unless parameters are fine-tuned, cortical neural networks dynamics lies in a relatively broad pseudo-critical region, where it is either subcritical or supercritical, a state termed *self-organized quasi-criticality* (Bonachela and Muñoz, 2010), or *extended criticality* (Longo et al., 2012), corresponding to Griffiths phases (Moretti and Muñoz, 2013). Accordingly, the “natural world” is presumably not “more critical” than the brain, a notion that would be inconsistent with a recently proposed mechanism of information transfer in complex systems (West et al., 2008; Aquino et al., 2011).

Furthermore, there is an essential contradiction in the suggestion that primary consciousness would be a “psychological atavism” and a “suboptimal mode” and, at the same time, “more critical” than normal states (p. 12). On the one hand, intuitively, the psychedelic state wouldn't appear to be functionally optimal. The hyper-connectivity which characterizes it (Tagliazucchi et al., 2016) is in general associated brain pathology (Whitfield-Gabrieli et al., 2009; Supekar et al., 2013; Hillary et al., 2015). During the psychedelic state the number of forbidden connectivity patterns may decrease, but some of these patterns may be associated with suboptimal (possibly even functionally detrimental) states. On the other hand, however, criticality has been shown to be optimal in terms of transmission and storage of information, computational capabilities, large network stability and sensitivity to sensory stimuli (Shew et al., 2009; Bonachela and Muñoz, 2010). In what sense can the brain be at the same time critical and suboptimal? And if primary consciousness is critical, and therefore in so many ways optimal, what's the evolutionary meaning of secondary states? A possible understanding of criticality and optimality of the psychedelic state is suggested by the results of Zare and Grigolini (2013) suggest. In this study, genuine temporal complexity was found to occur earlier than avalanche-size complexity, in a narrow range where information transfer becomes maximal. Interpreting this information transport enhancement as a signature of criticality, power law avalanches become a manifestation of supercriticality. Accordingly, normal waking and the psychedelic state may respectively lie in the critical and supercritical regimes.

## Conclusions

A true configurational entropy requires extensive phase space exploration, otherwise it merely reflects the random side of complexity. Under these conditions, quantifying the number of metastable states is not enough, *per se*, to assess criticality. Rather than just moving brain activity toward or away from criticality, context-specific demands can modulate the scaling properties of brain fluctuations (Popivanov et al., 2006; Bianco et al., 2007; Buiatti et al., 2007; Bhattacharya, 2009; Zilber et al., 2012), by modifying the scale at which they show scaling or by inducing transitions between different scaling regimes or cross-overs between universality classes (Papo, 2014).

## Author contributions

The author confirms being the sole contributor of this work and approved it for publication.

### Conflict of interest statement

The author declares that the research was conducted in the absence of any commercial or financial relationships that could be construed as a potential conflict of interest.
